# Presentation of multiple endocrine neoplasia type 2A-associated ectopic cushing’s syndrome: case report and a systematic review

**DOI:** 10.3389/fendo.2025.1644751

**Published:** 2025-11-11

**Authors:** Wei Wang, Wei-Ying Chen, Mei-Xian Zhang, Zhen-Yu Chen, Zhi-Lie Cao, Jun-Wei Wang, Wu-Gen Yao, Jian-Qiang Zhao, Fei-Ping Li, Hong-Yuan Yu, Jun Lu, Xiao-Ping Qi

**Affiliations:** 1 Department of Urology, Tiantai People’s Hospital of Zhejiang Province, Tiantai County, Taizhou, Zhejiang, China; 2 Department of Urology, Taizhou Hospital of Zhejiang Province Affiliated to Wenzhou Medical University, Enze Hospital of Hangzhou Medical College, Taizhou Enze Medical Center (Group), Taizhou, Zhejiang, China; 3 Evidence-based Medicine Center, Taizhou Hospital of Zhejiang Province Affiliated to Wenzhou Medical University, Taizhou, Zhejiang, China; 4 Department of Oncologic and Urologic Surgery, the 903rd People’s Liberation Army (PLA) Hospital, Hangzhou Medical College, Hangzhou, Zhejiang, China; 5 Department of Urology, Linghu People’s Hospital, Huzhou, Zhejiang, China; 6 Department of Head and Neck Surgery, Zhejiang Cancer Hospital, Hangzhou, Zhejiang, China

**Keywords:** multiple endocrine neoplasia type 2, medullary thyroid carcinoma, pheochromocytoma, Ectopic Cushing’s syndrome, hypercortisolism, RET proto-oncogene

## Abstract

**Background:**

Multiple endocrine neoplasia type 2 (MEN 2)-related ectopic Cushing’s syndrome (ECS) continues to present a clinical challenge due to its rarity and complexity. This study combines case analysis with a systematic literature review to elucidate the disease patterns.

**Summary:**

We present a 55-year-old male with MEN 2-associated ECS caused by metastatic medullary thyroid carcinoma (MTC) and review 21 literature cases. The mean age of ECS diagnosis was 37.0 years (range: 13-72), with a male predominance (64%). MEN 2A (16 cases) and MEN2B (6 cases) involved *RET* exons 10, 11, 16, with MEN2B patients developed ECS earlier than MEN 2A (*P* = 0.002). Of these, 14 presented ECS due to advanced-MTC (50% with distant metastasis), with the diagnosis of ECS following that of MTC in 57% of patients after an average interval of 72 months, while 43% had concurrent diagnoses. 7 were due to pheochromocytoma (PHEO), all presenting with concomitant diagnosis of PHEO and ECS, and 14% had metastasis. One case involved both PHEO and MTC. Severe hypercortisolemia and elevated adrenocorticotropic hormone were common. 64% of the 11 patients tested positive for adrenocorticotropic hormone (55%) or corticotrophin-releasing hormone (9%) immunostaining, while proopiomelanocortin mRNA or corticotropin-releasing factor/urocortin1/urocortin3 was detected in 2 others. Bilateral adrenalectomy (BLA, 13 patients) or unilateral adrenalectomy (1 patients) was performed in 14 out of 18 patients, with 83% of PHEO-related ECS achieving a cure, while advanced-MTC required multimodal therapy and 64% requiring eventual BLA treatment; One biphasic MTC/PHEO achieved good control. Evidence of tyrosine kinase inhibitors (TKIs) treatment for hypercortisolism in ECS and MTC remains limited. Mortality primarily resulted from ECS complications or MTC progression.

**Conclusions:**

MEN 2-related ECS should be considered in differentials. Adrenalectomy typically achieved cure in most ECS due to PHEO, but vigilance is required for the double risk of both hypercatecholaminemia and hypercortisolism during the perioperative period. Whereas most ECS due to advanced-MTC eventually required BLA to improve symptoms, yet prognosis remained generally poor. TKIs might offer benefits in the management of both MTC and hypercortisolism. The integration of *RET* testing, early diagnosis, and precise treatment can help prevent ECS complications and improve outcomes.

## Introduction

Multiple endocrine neoplasia type 2 (MEN 2) is a neuroendocrine cancer syndrome characterized by medullary thyroid carcinoma (MTC), which may or may not be accompanied by pheochromocytoma (PHEO), hyperparathyroidism (HPTH), and extraendocrine features (1–4). Clinically, MEN 2 presents with two distinct subtypes: MEN 2A (OMIM# 171400; ~95% of MEN 2 cases) and MEN 2B (OMIM# 162300; ~5%) (1). Nearly all cases of MEN 2 are caused by germline mutations in the REarranged during Transfection (*RET*) proto-oncogene (OMIM# 164761), leading to a gain of function (1–7). Approximately 95% of MEN 2A cases are associated with germline mutations in exons 10 and 11, whereas exons 16 and 15 are responsible for more than 95% of MEN 2B cases (1–5). Over the last three decades, management strategies for MEN 2 have evolved from clinical, evidence-based recommendations to an emphasis on *RET* risk category-specific molecular diagnosis. This shift has moved towards integrating predictive testing for molecular and biomarker-based precision diagnostic approaches to provide individualized clinical decision-making. Consequently, the clinical management of MEN 2 has undergone profound changes, and the prognosis for these patients has significantly improved (1–9). Recently, the successful application of preimplantation genetic testing for monogenic disorders and non-invasive prenatal sequencing has significantly contributed to effective pregnancy management and the prevention of MEN 2 and other human monogenic disorders (10–12).

It is intriguing that serum calcitonin (Ctn) and carcinoembryonic antigen (CEA) serve as the primary biomarkers for MTC. However, MTC can occasionally secrete elevated levels of bioactive hormones beyond Ctn/CEA, potentially resulting in a rare paraneoplastic syndrome. The most prevalent ectopic hormones are adrenocorticotropic hormone (ACTH) or corticotrophin-releasing hormone (CRH, <5%), which can cause excessive cortisol production and lead to Cushing’s syndrome. This condition is known as ectopic Cushing’s syndrome (ECS) or ectopic ACTH syndrome when it originates outside the pituitary (1, 13, 14). Additionally, the PHEO, a catecholamine-producing tumor, can sometimes secrete higher levels of ACTH or CRH than catecholamines, which may induce ECS (1, 15–19).

As of now, fewer than 200 cases of ECS secondary to MTC or PHEO have been documented in the literature (13–42). Nonetheless, occurrences of MEN 2-related MTC and/or PHEO leading to ECS are even less common, with the majority being case reports (20–42). In this paper, we present a *RET*-p.C634Y classic MEN 2A pedigree, an infrequently reported case of ECS, and conduct a systematic review of clinical data from other MEN 2 patients (families) with ECS previously reported. We aim to discuss the potential causes of ECS, evaluate the effectiveness of tumor debulking, steroidogenesis inhibitors, tyrosine kinase inhibitors (TKIs), bilateral adrenalectomy (BLA), and a new selective RET inhibitor in treating MEN 2-related MTC and ECS.

## Patients and methods

### Patient presentation

**Figure 1:**
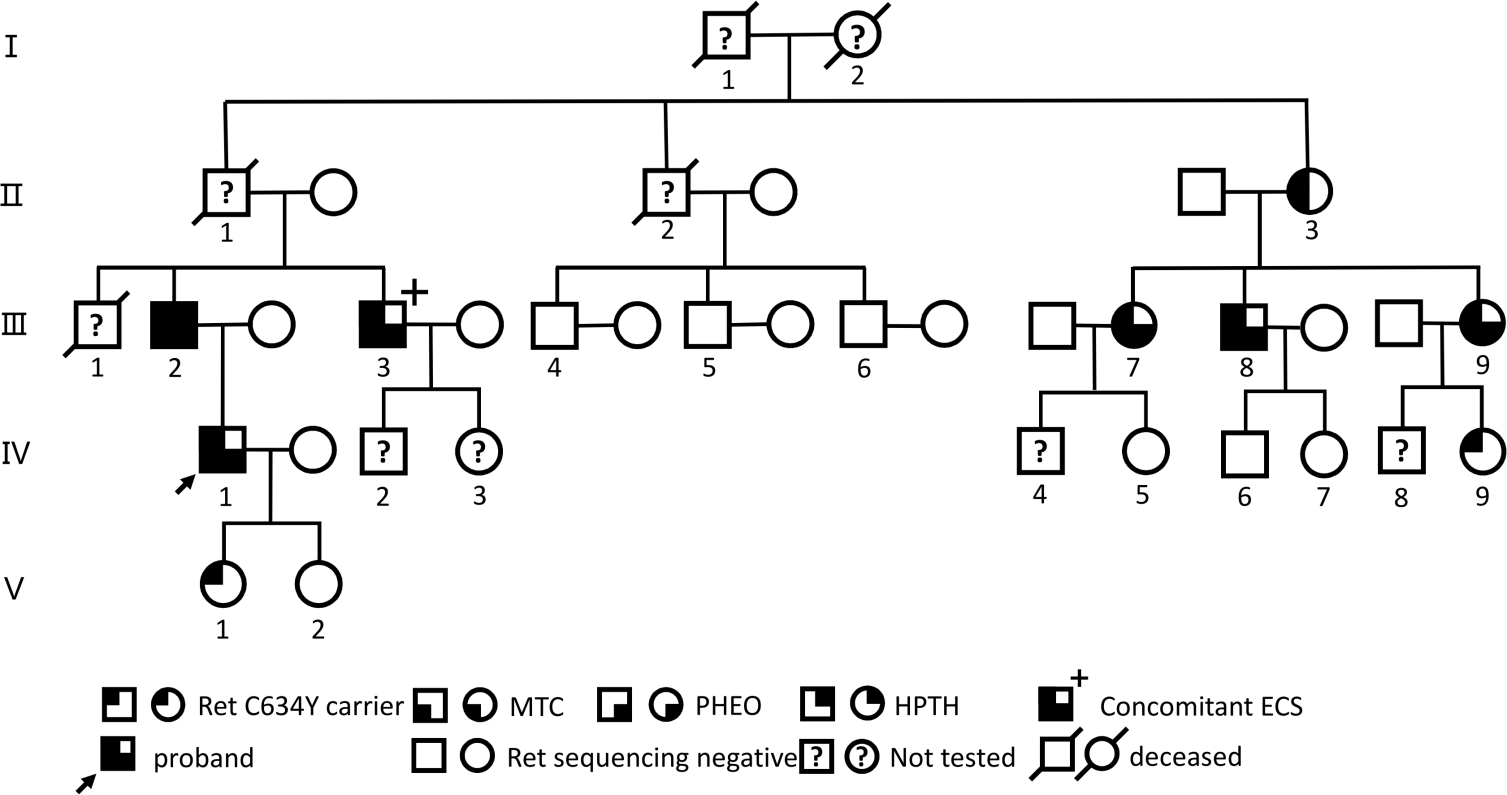
Genealogies of a family with the RET C634Y mutation and associated ECS. Numbers indicate family members. Circles and squares indicate females (F) and males (M), respectively. MTC, medullary thyroid carcinoma; PHEO, pheochromocytoma; HPTH hyperparathyroidism; ECS, ectopic Cushing’s syndrome.

In July 2022, a 54-year-old male (III-3; **Figure 1**, **Supplementary Table S1**) was admitted to the local hospital of traditional Chinese medicine with a 2-year history of bilateral palpable neck mass. Both ultrasound and computed tomography (CT) scans revealed bilateral thyroid masses (left, 2.3 cm; right, 4.8 cm; **Supplementary Figures S1A, B**). A ultrasound-guided fine-needle aspiration was performed on both thyroid nodules, and cytological examination showed features positive for malignancy, suggesting possible MTC. Subsequently, he underwent a total thyroidectomy with bilateral level VI lymph node dissection. Bilateral multifocal MTC with bilateral lymph node metastasis (LN+/resected, 6/6) was confirmed by histopathological examination. Post-operative serum Ctn levels were 1358.4 pg/ml (normal range for males, < 8.4; females, < 5.0). In March 2023, the patient experienced generalized fatigue, and serum levels of potassium and calcium decreased to 2.9 mmol/L and 1.45 mmol/L, respectively, while Ctn >2000pg/ml. Oral and intravenous potassium and calcium supplements struggled to restore these levels to normal. Following the diagnosis of recurrent right MTC with bilateral neck and mediastinal lymph node metastasis by CT imaging (**Supplementary Figures S1C–F**) and fine-needle aspiration at an external hospital, he underwent surgery for the right residual thyroid with modified bilateral lateral neck and mediastinal lymph node dissection. Pathological results confirmed right MTC with bilateral lateral neck and mediastinal lymph node metastasis [LN+/resected, 45/67; T3aN1bMx (43)]. Postoperative Ctn levels decreased to 1853.0 pg/ml.

In September 2023, his blood pressure fluctuated within the range of 166–180/95–110 mmHg, and his blood glucose levels reached 17.6 mmol/L. After treatment with amlodipine besylate 5 mg twice daily, irbesartan 150 mg once daily, metformin 0.5 g twice daily, and selegiline 0.1 g once daily, his blood pressure remained high at 150/100 mmHg, and his blood glucose levels fluctuated between 10.51 and 18.38 mmol/L. Neither treatment was effective.

In October, he was referred to our hospital. A physical examination revealed a Cushingoid appearance, polycythemia, skin thinning, a full moon face, a buffalo hump, and hypertension with readings of 200/95 mmHg. Biochemical evaluation showed markedly elevated plasma ACTH and serum cortisol levels at 8:00 am, 4:00 pm, and 12:00 pm, elevated 24-hour urinary free cortisol (1200 μg/24h; reference range, 58.00-403.00), elevated fasting blood glucose, and significantly low serum potassium and calcium. Ctn was >2000 pg/ml and CEA was 70.8 ng/mL, plasma metanephrine was slightly increased, while normetanephrine and 3-methyltyramine levels were within normal limits. There was a loss of diurnal cortisol and ACTH variation, failure to be suppressed by 1 mg dexamethasone, and lack of response to CRH (**Supplementary Table S2**). No pituitary adenomas were revealed by enhanced magnetic resonance imaging (MRI), but CT/18F-FDG-PET/CT imaging disclosed multiple metastatic lesions from MTC in the neck, bilateral adrenal hyperplasia-like changes, and scattered infectious lesions in both lungs and the left iliac bone with slightly increased density. A suspected diagnosis of “MEN 2A-related ECS” was made. He received anti-infective treatment, low molecular weight heparin for anticoagulation, intensive glycemic control using an insulin subcutaneous pump, and multiple oral medications to manage blood pressure. Additionally, he was prescribed oral spironolactone (up to 100 mg/day), daily intravenous potassium supplementation exceeding 10 grams, intravenous calcium supplementation, and oral activated vitamin D. Despite these interventions, glucose and blood pressure control remained abnormal, and the persistent, intractable hypokalemia and elevation of calcium levels were not significantly improved. Following a multidisciplinary team discussion and based on the recurrence of MTC cytoplasmic negativity for CRH, and positivity for Ctn, CEA, weak positivity for ACTH as shown by immunohistochemical staining (**Supplementary Figures S2A, B**), he was diagnosed with “MEN2A-related MTC with ECS.” Subsequently, he underwent laparoscopic BLA via the transperitoneal approach and received an appropriate dose of hydrocortisone replacement, as guided by previous reports (44). The pathological results indicated bilateral adrenal cortex hyperplasia and a tiny PHEO (1.2 cm) (**Supplementary Figures S3A, B**), while being positive for chromogranin A, synaptophysin, neuronspecific enolase but negative for ACTH, CRH, and Ctn, CEA by immunohistochemical staining. He was discharged from the hospital one week after the BLA procedure. No Clavien-Dindo graded surgical complications occurred. At postoperative months 1, 3, 6, and 12, his hypercortisolism had completely resolved, and medications for ECS-related hypertension and hypoglycemia were gradually withdrawn, even though cortisol concentrations had risen again 12 months after the BLA. Despite the ongoing progression of MTC, levels of Ctn remained consistently above 2,000 pg/mL, CEA ranged from 77.1 to 880.5 ng/mL, and ACTH levels showed a steady increase from 183.0 to 649.58 pg/mL (**Supplementary Table S2**). Additionally, imaging by emission CT and/or MRI revealed enlarged bilateral neck lesions, multiple metastases in the ribs, shoulder blades, vertebrae, and bilateral pelvis, as well as recent liver metastases with a maximum diameter of 1.6 cm (T3aN1bM1). Despite this, he continued treatment with a clinical trial of a selective RET inhibitor, the drug code HS-10365 (protocol No., HS-10365-101; Jiangsu Haosun Pharmaceutical Group Co., Ltd.), administered at a dosage of 160 mg, twice daily, orally, starting one month after the BLA.

Before and after the patient’s treatment at our hospital, a five-generation family pedigree analysis was conducted, as previously reported in our study (44, 45). Overall, out of 16 individuals, 9 were found to carry the *RET*-p.C634Y (c.1901G>A) germline mutation through targeted sequencing from March 2023 to February 2024 (**Figure 1**, **Supplementary Table S1**). Among these, 7 presented with MEN 2A-related MTC, PHEO, and/or HPTH, with 1 individual (III-3) having MTC-related ECS; 6 of the 7 underwent surgery, including 1 (III-9) who underwent a sequential surgical treatment for PHEO and MTC, while 1 (II-3) refused any treatment. Of the remaining 2 carriers, 1 (IV-9) declined further examination and 1 (V-1) opted for a watchful waiting approach. Nevertheless, the patient with ECS (III-3) exhibited a more advanced TNM staging of MTC and higher post-Ctn levels compared to other carriers without ECS within this family.

### Systematic review of the literature

Search Strategy and Data Collection Criteria: A systematic review was conducted to identify pedigrees and patients with MEN 2-related ECS, synthesizing patient clinical characteristics, treatment strategies, and disease outcomes. This review adhered to the Preferred Reporting Items for Systematic Reviews and Meta-Analyses (PRISMA) guidelines (46).

Two reviewers (W.W., M.X.Z.) independently searched the electronic databases PubMed, Web of Science, Scopus, and EMBASE for publications up to November 8, 2024. The literature retrieval strategies employed the following Title and Abstract terms: (“multiple endocrine neoplasia type 2” OR “multiple endocrine neoplasia type II” OR “MEN 2” OR “MEN II” OR “multiple endocrine neoplasia” OR “medullary thyroid carcinoma” OR “pheochromocytomas”) AND (“ACTH” OR “cushing syndrome” OR “paraneoplastic syndrome” OR “paraneoplastic” OR “hypercortisolism” OR “ectopic cushing”). No language and time restrictions were applied. To identify relevant articles not already found in this search, we also examined the references of retrieved manuscripts and included relevant studies.

Full texts of articles employing the Joanna Briggs Institute (JBI) critical appraisal checklist for case series and case reports were evaluated (47). Publications unrelated to humans or duplicates were immediately discarded. Subsequently, two reviewers (W.W., M.Z.) independently screened each title and abstract to select articles for full-text review and to determine their eligibility for the study. All articles reporting cases (pedigrees) of MEN 2-related ECS were considered relevant, and studies providing data on individual participants were also included. Disputes were resolved through consensus among all authors. No study was excluded based on quality, as the available evidence was extremely limited.

Data extraction and verification. For each included article, available data, including patient demographics, clinical characteristics, biochemical information, endocrine tumor status and treatment, ECS treatment, immunohistochemistry results, and disease outcomes, were extracted independently by two reviewers (W.W., M.X.Z.). A cross-check was then performed. Any discrepancies were discussed and rechecked, and verified by a third reviewer (X.P.Q.).

Due to the significant heterogeneity of the included studies, we were unable to perform a meta-analysis and conducted only a narrative synthesis.

### Statistical analysis

Statistical analyses were conducted using SPSS^®^, version 25.0 (IBM, Armonk, NY, USA) for data processing. Continuous variable data was summarized as mean ± standard deviation (Mean ± SD) or presented as the median (interquartile range [IQR] or range). The Fisher’s exact or the exact Mann–Whitney–Wilcoxon rank sum test was used for intergroup comparisons of categorical data, as appropriate. Statistical significance was set at *P* < 0.05.

## Results

### Included articles

**Figure 2:**
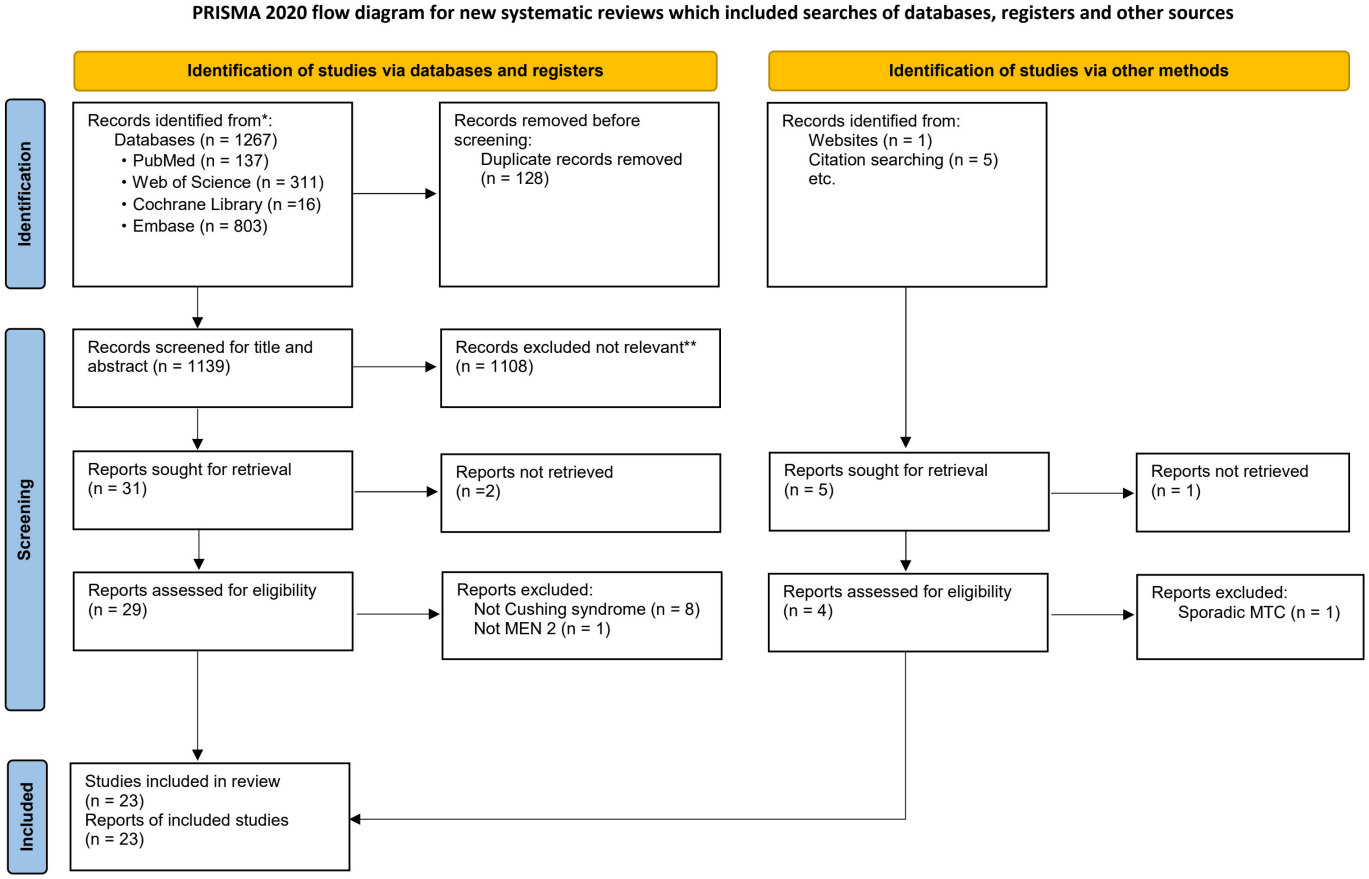
Flowchart of literature search and study selection. Adapted from PRISMA. *Consider, if feasible to do so, reporting the number of records identified from each database or register searched (rather than the total number across all databases/registers). **If automation tools were used, indicate how many records were excluded by a human and how many were excluded by automation tools. PRISMA, Preferred Reporting Items for Systematic Reviews and Meta-Analyses; MEN 2, Multiple Endocrine Neoplasia type 2. *From:* Page MJ, McKenzie JE, Bossuyt PM, Boutron I, Hoffmann TC, Mulrow CD, et al. The PRISMA 2020 statement: an updated guideline for reporting systematic reviews. BMJ 2021;372:n71. doi: 10.1136/bmj.n71.

After eliminating duplicates, a total of 1,273 articles were identified for screening. Out of these, 36 were selected for assessment following a review of their titles and abstracts. After a full-text review, 3 articles were excluded due to extremely rare and/or unavailability of clinical information, 10 articles were excluded because they involved patients with non-Cushing’s syndrome, non-MEN 2 or sporadic MTC. Ultimately, 23 articles containing data on 21 patients with MEN 2-related ECS from 21 unrelated MEN 2 pedigrees were included (**Figure 2**).

### Pedigrees and patients’ characteristics

The clinical presentation of our case, with detailed information, is depicted in **Supplementary Table S1 and Table S2**. A summary of 22 patients, including this case and 21 previous ones, is presented in **Tables 1**–**3**. There were 16 cases of MEN 2A (73%) and 6 cases of MEN 2B (27%). The mutations were located in exons 11/10 in 8 cases of MEN 2A [38%; exon 11 (19%; C634) and exon 10 (19%; C618/611/609)], in exon 16 in 3 cases of MEN2B (14%; M918T), and the mutation type was unavailable for the remaining 11 patients [50%; 8 MEN 2A (36%) and 3 MEN 2B (14%)]. Among these 22 patients, 14 (64%) had ECS due to MTC, 7 (32%) due to PHEO, and 1 (4%, ID:22) due to both MTC and PHEO (the MTC-to-PHEO ratio was 2:1) (**Table 1**).

**Table 1:** Clinical characteristics in patients with MEN 2 at the time of diagnosis of ECS.

^#^Patient ID number	RET mutation/MEN 2 (A/B)	Present (^†^initial) age (years)	Sex (M/F)	Presentation	MTC			PHEO status (maximum diameter, cm)	PHPT status	IHC analysis for MTC/PHEO		References
					Stage at diagnosis	Site of metastasis (from initial treatment of MTC)	Pre/Post-Ctn (pg/mL) (normal range)			ACTH	CRH	
1	NA/MEN2A	46 (46)	F	Weakness,clumsiness, hypertension and CA	M1	LN+,lung, pleura, pericardium, liver, ovarie, adrenal, small intestine	NA	Bilateral (20)	+	NA	NA	Williams et al. (20)
2	NA/MEN2A	45 (42)	M	NA	LN+	LN, liver (25 months)	2680/NA (<10)	−	−	NA	NA	Mure et al. (21)
3	NA/MEN2B	20 (20)	F	Tongue and eyelid mucosal neuromas, thyroid enlargement, CA	LN+, infiltrating the tracheal	left anterosuperior mediastinal, lung (12 months)	10000/9000 (<20-95)	−	−	−	+/MTC	Tagliabue et al. (22)
4	C611/MEN2A	47 (36)	M	Hypertension, weight gain, CA	Local	LN, Liver, bone (132 months)	NA/24000 (< 8.8)	−	−	−	NA	von Mach et al. (23)
5	C618R/MEN2A	34 (30)	M	Weakness, bruising, hyperdefecation,skin rash, multiple rib fractures, CA	LN+	LN	NA/2500~4700 (NA)	Right (2.0)	+	^§^ −	NA	Smallridge et al. (24)
6	NA/MEN2A	45 (43)	M	NA	LN+, lung+	LN, lung, bone, liver	NA/>2000(< 10)	NA	NA	NA	NA	Barbosa et al. (25)
7	C609Y/MEN2A	51 (51)	F	Weight gain, fatigue, abdominal striae, diabetes, hypertension, amenorrhea	Metastatic	Live (concomitant)	698/NA (< 4.7)	−	−	NA	NA	Zaydfudim et al. (26)
8	NA/MEN2B	13 (13)	M	Growth retardation, inability to walk, CA, Marfanoid habitus	LN+	LN (concomitant)	1100/NA (< 19)	−	−	−	NA	Dhanwal et al. (27)
9	NA/MEN2B	31 (11)	F	Hyperpigmentation, multiple lingual tumors, pachycheilia	Local	Liver (96 months), brain, bone (120 months)	NA/28125 (< 100)	Bilateral (NA)	−	NA	NA	Kurozumi et al. (28)
10	M918T/MEN2B	17 (17)	M	Centripetal obesity, striae, hypertension, bruising, severe acne, infections, muscle weakness	Metastatic	LN, lung, bone, left adrenalparenchyma, periadrenals of tissue	2000/1683 (< 16)	−	−	+/MTC	NA	Fox et al. (29) Nella et al. (30)
11	M918T/MEN2B	13 (10)	M	Growth deceleration, weight gain, lips/tongue neuromas, fractures	Metastatic	LN, lung (24 months, S), lung, bone (33 months)	4658/1688–6003 (<6)	−	−	+/MTC	NA	Singer et al. (31)
12	C618Y/R844Q/ MEN2A	72 (72)	M	Hypertensive crisis, muscle weakness, pain, hyperpigmented, weight and body hair loss	Metastatic	Metastatic	7330/NA	−	−	NA	NA	Ares et al. (32)
13	NA/MEN2A	52 (47)	M	Neck mass	Metastatic	LN, liver, and bone	NA	NA	NA	NA	NA	Agosto et al. (33)
14	C634Y/MEN2A	56 (54)	M	CA,hypertension, full moon face, buffalo hump and diabetes	LN+	LN, bone, liver (18 months)	NA/>2000 (<8.4)	−	−	+/MTC	−	**Current study**
15	C634R/V648I /MEN2A	34 (34)	M	CA and bilateral adrenal nodules	N0	−	4660/<50 (<50)	Bilateral (NA)	−	NA	NA	Mendonça et al. (34) Nunes et al. (35)
16	NA/MEN2A	26 (26)	F	CA	NA	NA	NA	Bilateral (NA)	NA	NA	NA	Ferna´ndez-Cruz et al. (36)
17	NA/MEN2A	69 (69)	F	Fever, drowsiness, watery diarrhea and hyperglycemia	−	−	NA	Left (NA)	+	+/PHEO	NA	Nozawa et al. (37)
18	C634R/MEN2A	29 (29)	M	Hypertension, headache, hyperglycemia.	N0	N0M0	116.40/NA (< 10)	Bilateral (NA)	+	+/PHEO	−	Moon et al. (38)
19	+/MEN2A	26 (26)	F	Fatigue, numbness in the limbs, muscle weakness, skin lesions, headache, hypertension, CA, hyperpigmentation	NA	NA	120/NA (< 14)	Bilateral (3.5)	NA	NA	NA	Borzouei et al. (39)
20	M918T/MEN2B	28 (28)	M	Hypertension, diabetes, marfanoid features and bumpy lips	NA	NA	1649/increased (<18.2)	Bilateral (LN+, M1)	−	+/PHEO	−	Kruljac et al. (40)
21	NA/MEN2A (s)	23 (23)	M	Abdominal mass	NA	NA	15.2/NA (<8.4)	Right (12.7)	+	NA	NA	Shah et al. (41)
22	C634R/MEN2A	38 (38)	F	Evaluation of bilateral adrenal incidentalomas	LN+ (s)	LN (concomitant, s)	> 1600/NA	Bilateral (NA)	−	^*^ −	NA	Kageyama et al. (42)

MEN 2 (A/B), multiple endocrine neoplasia type 2 (A/B); ECS, ectopic Cushing’s syndrome; M/F, male/female; MTC, medullary thyroid carcinoma; post-Ctn, post-surgery basal serum calcitonin; PHEO, pheochromocytoma; PHPT, primary hyperparathyroidism; IHC, Immunohistochemical; ACTH, adrenocorticotropic hormone; CRH, corticotropin releasing hormone; CA, Cushingoid appearance; N0, negative for lymph nodes metastases; LN+, positive for lymph nodes metastases; LN, lymph nodes; -, negative; NA, not available; s, suspicion.

^#^, Patient ID 1~14 had ECS due to MEN 2-related MTC; patient ID 15~21 had ECS due to MEN 2-related PHEO; patient ID 22 had ECS caused by MEN 2-related MTC and PHEO.

^†^, The initial diagnosis age of the corresponding diseases that cause ECS.

^§^, Proopiomelanocortin (POMC) mRNA was detected by *in situ* hybridization (ISH) in the MTC tissue, but not in the PHEO, and not ACTH by immunostain in the MTC.

^*^, CRF, Ucn1, and Ucn3 immunoreactivity was strongly detected (+++) in almost all tumor cells of PHEO, while moderate positive (++) in cells of MTC, whereas ACTH immunoreactivity was absent in all the PHEO and MTC.

**Table 2:** Demographics and presentations of hypercortisolism at time of diagnosis in patients with MEN 2 and ECS (references to 2 major published studies by Corsello et al. (14) and Kishlyansky et al. (15) reported).

Patient characteristics	Patients with MEN 2 and ECS (^†^n = 21)		Patients with MTC and ECS	Patients with PPGL and ECS
	MTC with ECS (n =14)	PHEO with ECS (n = 7)	(^#^n = 86 (14)	(^*^n = 94 (15)
Age at initial disease diagnosis, years				
Mean (SD)	35.1 (18.9)	33.6 (16.0)	44.4 (15.3)	NA
Median (IQR)	39 (16-48)	28 (26–34)	45 (34-54)	47 (NA)
Range	10–72	23–69	10–84	10–80
Age at the time of ECS diagnosis, years				
Mean (SD)	38.7 (17.9)	33.6 (16.0)	47.7 (16.1)	NA
Median (IQR)	45(19.3–51.3)	28 (26–34)	49.0 (36.2-58.0)	47 (NA)
Range	13–72	23–69	10–78	10–80
Sex				
Male	10 (71.4%)	4 (57.1%)	55 (64%)	26 (28%)
Female	4 (28.6%)	3 (42.9%)	31 (36%)	68 (72%)
Manifestations of hypercortisolism ^§^				
Cushingoid appearance	10 (83%)	4 (67%)	48 (56%)	67 (82%)
Central obesity	5 (42%)	1 (25%)	26 (30%)	NA
Moon face	6 (50%)	1 (25%)	32 (37%)	NA
Buffalo hump	5 (42%)	1 (25%)	17 (20%)	NA
Hyperpigmentation	3 (25%)	1 (25%)	17 (20%)	NA
Diabetes mellitus	4 (33%)	3 (75%)	33 (38%)	36 (63%)
Hypertension	10 (83%)	3 (75%)	37 (42%)	64 (86%)
Muscle weakness	8 (67%)	1 (25%)	36 (41%)	NA
Mood alteration	2 (17%)	1 (25%)	10 (12%)	27 (29%)
Hirsutism	3 (25%)	NA	11 (13%)	NA
Striae (Purple striae)	6 (50%)	NA	17 (20%)	NA
Osteoporosis (fracture)	3 (25%)	NA	16 (19%)	8 (9%)
Infections	3 (25%)	1 (25%)	NA	22 (23%)
Diarrhea	2 (17%)	1 (25%)	23 (27%)	NA
Bruising	4 (33%)	NA	18 (21%)	NA
Growth retardation	2 (17%)	NA	NA	NA
Hypokalemia	4 (33%)	1 (25%)	NA	38 (62%)
Increased plasma ACTH levels	12 (100%)	2 (50%)	NA	63 (88%)

MEN 2, multiple endocrine neoplasia type 2; ECS, ectopic Cushing’s syndrome; MTC, medullary thyroid carcinoma; PHEO, pheochromocytoma; PPGL, PHEO and paraganglioma.

(PGL); SD, standard deviation; IQR, interquartile range; ACTH, adrenocorticotropic hormone.

**^†^**All 22 patients with MEN 2 and ECS in addition to a 38-year-old MEN 2A with ECS due to MTC and PHEO.

**^#^**86 patients all with MTC include 10 hereditary or 1 probably hereditary MTC.

^*^94 patients all with PPGL including 74 PHEOs and 20 PGLs.

**^§^**12 out of 14 patients with ECS due to MTC and 4 out of 7 patients with ECS due to PHEO in the presence of patients with relatively detailed information on hypercortisolism.

**Table 3:** Treatment and outcome in patient with MEN 2 and ECS.

^#^ Patient ID number	Time between MTC and ECS diagnosis (months)	Treatment of MEN 2 related MTC or PHEO	Treatment of ECS	Follow-up from treatment of ECS (Months)	Outcome	References
1	Concomitant	None	None	0	Died during laparotomy	(20)
2	29 months (MTC before ECS)	TT + RLND + RT + CHT	SSA **→** ketoconazole **→** mitotane **→** BLA	10	Die of MTC	(21)
3	Concomitant	TT + RLND	TT + RLND	12	Survival	(22)
4	132 months (MTC before ECS)	TT + PTD	BLA	NA	Survival	(23)
5	48 months (MTC before ECS)	TT + PTD + RA + RLND (in two times)	BLA	NA	NA	(24)
6	17 months (MTC before ECS)	NA	NA	29	Died of MTC	(25)
7	Concomitant	CHT (octreotide → 5-flourouracil → streptozosin)	Ketoconazole → a Phase II tyrosine kinase inhibitor trial + BLA	NA	Survival	(26)
8	Concomitant	TT + RLND	TT + RLND	1	Survival	(27)
9	240 months (MTC before ECS)	TT + Brain metastasis resection + BLA (for PHEO 36 months before ECS)	NA	NA	Survival	(28)
10	Concomitant	TT + RLND + metastasectomy + RT + vandetanib → sunitinib → sorafenib → cabozantinib	Vandetanib → ketoconazole → BLA	72	Died of MTC	(29, 30)
11	33 months (MTC before ECS)	TT + RLND	BLA	NA	NA	(31)
12	Concomitant	TT + RLND	Ketoconazole + TT + RLND	NA	NA	(32)
13	60 months (MTC before ECS)	TT + RLND + vandetanib → cabozantinib + RT on bone metastases	Metyrapone → selpercatinib	NA	NA	(33)
14	15 months (MTC before ECS)	TT + RLND (in two times)	BLA + a Phase I/II, highly selective RET kinase inhibitor (HS-10365) trial	12	Survival	Current study
15	Concomitant	TT + RLND	BLA	108	Died of myocardial ischemia	(34, 35)
16	NA	NA	BLA	NA	Survival	(36)
17	None	NA	None	0	Died of ECS	(37)
18	Concomitant	BLA+ TT + a right lateral selective LND, and PTD (2/4)	BLA	0.4	Survival	(38)
19	4 months (ECS before MTC)	BLA + TT	BLA	6	Survival	(39)
20	Concomitant	TT + total colectomy + radical BLA with lymph node metastasectomy (single-stage)	Radical BLA with lymph node metastasectomy	NA	Survival	(40)
21	Concomitant	RA	RA	NA	NA	(41)
22	Concomitant	BLA + TT + PTD	BLA + TT	NA	NA	(42)

MEN 2, multiple endocrine neoplasia type 2; ECS, ectopic Cushing’s syndrome; MTC, medullary thyroid carcinoma; BLA, Bilateral adrenalectomy; TT, total thyroidectomy; RLND, regional lymph node dissection (distinction between central, laterocervical and mediastinal lymph node dissection in most studies was not available); RT, radiotherapy; CHT, chemotherapy; SSA, somatostatin analogues; PTD, Parathyroidectomy; RA, Right adrenalectomy; PHEO, pheochromocytoma.

^#^, Patient ID 1~14 had ECS due to MEN 2-related MTC; patient ID 15~21 had ECS due to MEN 2-related PHEO; patient ID 22 had ECS caused by MEN 2-related MTC and PHEO.

### ECS in MEN2-related MTC or PHEO

There were a total of 14 males and 8 females, resulting in a male-to-female ratio of 1.8:1. The mean age at diagnosis of ECS was 37.0 years (range, 13-72) (**Table 1**). With one exception (ID:22), the mean age at diagnosis of ECS in 14 patients with MTC was not significantly different from that in 7 patients with PHEO (38.7 ± 17.9 years versus 33.6 ± 16.0 years; *t* = 0.64, *P* = 0.53). However, the mean age at diagnosis of ECS was significantly different between patients with MEN 2A and those with MEN 2B (43.3 ± 14.7 years versus 20.3 ± 7.6 years; *t* = 3.62, *P* = 0.002). The frequency of ECS due to MTC/PHEO in patients with MEN 2A did not significantly differ from that in patients with MEN 2B (10/5 versus 5/1; *P* = 0.62; **Tables 1**, **2**). Unfortunately, detailed information on hypercortisolism was available for only 16 patients, including 2 with subclinical Cushing’s syndrome (ID:21) or atypical Cushingoid appearance (ID:22) (**Tables 1**, **2**).

Among the 14 cases of MTC that resulted in ECS, the most prevalent clinical presentation at diagnosis was hypertension, muscle weakness, striae (purple striae), moon face, and/or symptoms indicative of ECS. At the time of diagnosis, all 14 patients exhibited metastatic disease; 7 (50%) presented with significantly elevated Ctn and distant metastasis (lung, liver, bone). Furthermore, 5 (36%) had lymph node metastasis, and 2 (14%) had local or locally advanced disease. The diagnosis of ECS was made subsequent to MTC in 8 out of 14 patients (57%), with a mean interval of 72 months (range, 15-240), and was concurrent in 6 out of 14 patients (43%; **Tables 1**–**3**).

Regarding the seven cases of PHEOs associated with ECS, the primary manifestations included hypertension, hyperglycemia, muscle weakness, and a Cushingoid appearance, with bilateral predominance observed in 71% (5/7) of cases. All 7 (100%) were simultaneously diagnosed with PHEO and ECS. Notably, only one (14%) exhibited lymph node metastasis of PHEO. Interestingly, the remaining case (ID:22), was suspected to have biphasic MTC and PHEO, leading to atypical CS. The age at diagnosis was 38 years (**Tables 1**–**3**).

MEN 2-associated ECS cases were almost always reported as individual instances, and the lack of clinical information on family members, particularly those from the same generation, complicates the comparison of the unique clinical features of MEN 2 patients with or without ECS within their respective families.

### Biochemical and immunohistochemical results

As anticipated, 12 out of 14 patients with ECS due to MTC exhibited significantly elevated Ctn levels (at least >500pg/mL) both pre- and post-operatively. Additionally, 2 cases were unavailable for analysis. This corresponds to the advanced stage of MTC. In contrast, at least 4 out of 7 patients with ECS due to PHEO showed only a slight increased Ctn levels (< 150pg/mL) pre- and post-operatively, indicating a relatively low clinical staging of MTC. Apparently, most patients with MEN 2 and ECS exhibited severe hypercortisolism, with a significantly increased plasma ACTH (88%, 14/16) and cortisol (92%, 12/13), as well as in 24-hour urinary-free cortisol (87%, 13/15). Cortisol was not suppressed by low/high-dose dexamethasone in 13 out of 14 patients (93%), and there was an absence of response to CRH in 2 out of 2 patients (100%). Regarding the identification of the source of ACTH and/or CRH, 11 out of 22 patients were evaluated. Among these, 7 patients with MTC and ECS, 3 patients (43%) exhibited positive ACTH immunoreactivity in MTC cells, while 4 showed negative expression. However, 1 of the latter had positive CRH staining, and another had proopiomelanocortin mRNA expression. Among the remaining 3 patients with PHEO and ECS, all (100%) displayed positive expression for ACTH in PHEO cells. In total, positive ACTH or CRH immunostaining was detected in 7 out of 11 patients (64%). The remaining 1 patient (ID:22), who had biphasic MTC/PHEO, expressed negatively for ACTH but positively for corticotropin-releasing factor (CRF), urocortin 1, and urocortin 3. Although plasma ACTH levels and their immunohistochemistry were normal or unavailable in a small number of patients with MEN 2, after the resection of MTC and/or PHEO, or with drug control, when their hypercortisolemia is alleviated and no other ectopic tumors are detected, ECS should be considered to originate from MTC and/or PHEO (**Table 1**).

### Treatment and outcome

In total, 16 patients (73%) underwent a total thyroidectomy, with 11 also undergoing regional lymphadenectomy. Out of these, 5 had only total thyroidectomy. Among the remaining 6 patients (27%), 1 received systemic chemotherapy alone, while the treatment details for the other 5 were not specified (**Table 3**). Post-surgery, one patient underwent neck radiotherapy and chemotherapy, another received radiotherapy and four TKI drugs, and a third was treated with two TKI drugs and radiotherapy for bone metastases.

Treatment information for ECS was available for 18 patients, while it was unavailable for the remaining 4 patients (including 3 with MTC and 1 with PHEO-associated ECS). Two of these patients died from ECS-related complications or underwent laparotomy without additional therapy (**Table 3**).

Of these, 11 out of 18 patients (61%) with ECS due to MTC experienced a successful resolution of hypercortisolism. Among 4 patients (36%) who did not undergo BLA: 2 patients who underwent total thyroidectomy with regional lymphadenectomy, followed by 1 successive treatment with metyrapone and selpercatinib, and 1 patient treated with ketoconazole followed by total thyroidectomy and regional lymphadenectomy, respectively. The remaining 7 patients (64%), BLA was used as the first-line treatment for ECS in 4 patients, where they had uncontrollable hypercortisolism, compression fractures, concomitant PHEO, and absence of a site of paraneoplastic ACTH secretion. For the other 3 patients, BLA served as a rescue or salvage treatment following the failure of neck radiotherapy, chemotherapy, steroidogenesis inhibitors (such as ketoconazole), and/or TKIs (1 on Vandetanib and 1 on a Phase II TKI trial). Achieving good control of ECS due to advanced-MTC proved to be challenging or, in some cases, impossible.

On the other hand, 6 out of 18 patients (33%) with ECS due to PHEO, BLA was used as the exclusive treatment for bilateral PHEO and ECS in 5. Consequently, remission of hypercortisolism and hypertension was achieved, whereas the control of the condition was unclear in 1 (ID:21), who underwent a right adrenalectomy for right PHEO and ECS. The BLA procedure was more effective in eliminating hypercortisolism caused by PHEO-related ECS than by MTC-related ECS. Additionally, in the case of one patient (ID:22) (6%), only the combination of BLA and total thyroidectomy achieved good control of hypercortisolism.

Information on survival was extremely limited, with only 16 cases reported, including 6 deaths (range, 0–108 months). Despite the use of various comprehensive treatment approaches, the prognosis for ECS resulting from MTC was often poor, primarily associated with ECS complications or MTC progression. In contrast, ECS due to PHEO may achieve good control or be cured by adrenalectomy (**Table 3**).

## Discussion

Here, we describe a family affected by MEN 2A, consisting of 9 individuals who carried the *RET*-p.C634Ymutation. Among them, a 55-year-old male presented with severe and refractory hypercortisolism due to ECS caused by metastatic MEN 2A-related MTC (T3aN1bM1). After extensive surgery to remove MTC and BLA, along with a trial of a highly selective RET inhibitor, his hypercortisolemia was rapidly resolved. However, 12 months after BLA, his cortisol levels increased again, and the MTC continued to progress, suggesting a more limited prognosis. Considering the current case, family history, and our previous reports, cases involving MTC or PHEO should be scrutinized for *RET* mutations (1–3, 17), and the cause of ECS was found to be less than 0.4% in 285 cases with MEN 2 (48).

Our systematic review has compiled findings that disclose the clinical characteristics of a total of 22 patients. There is a male predominance (1.8:1), and the mean age at diagnosis is 37 years, which is younger than that of previously reported cases of ECS due to MTC or PHEO (13, 14, 16, 25). The ratio of MEN 2A-to-MEN 2B was 2.7:1, involving exons 10, 11, and 16 of *RET*. However, the proportion of patients affected by the MEN 2B form was higher compared to its incidence, which is approximately 95%: 5% (19:1) (1–3). As anticipated, the mean age at diagnosis for ECS in MEN 2B patients was younger than in those with MEN 2A (*P* = 0.002), with a MTC-to-PHEO ratio of 2:1. However, the frequency of ECS resulting from MTC/PHEO in patients with MEN 2A or MEN 2B did not vary significantly (*P* = 0.62), and there was a bilateral PHEO predominance (72%). This indicates that the onset of ECS is in line with the natural evolutionary characteristics of MEN 2A and MEN 2B (1–3).

Of these 14 individuals who presented with ECS due to MTC, 57% had MTC precede the ECS diagnosis by 72 months, while 43% were diagnosed with both conditions simultaneously. The diagnosis of ECS-related hypercortisolism generally coincided with the onset of metastatic lung, liver, and bone disease, indicating a more aggressive biological behavior and advanced-MTC compared to that of MTC not associated with paraneoplastic syndrome (13, 14, 17, 25). Meanwhile, among the other 1 in 7 (14%) individuals with ECS due to PHEO, distant metastasis was present at diagnosis (40). In contrast, the prevalence of distant metastasis in PHEO associated with MEN 2 is less than 1% (6). The relatively high prevalence of distant metastasis suggests that ACTH or CRH secretion might start with the onset of the disease. As the tumor burden increases or the tumor microenvironment changes, tumor cells may dedifferentiate (14, 49), and the ability to produce ACTH or CRH may accumulate, leading to clinically significant hypercortisolism (14, 17). Unexpectedly, the individual with ECS due to both MTC and PHEO exhibited increased levels of plasma cortisol and CRF, yet had normal ACTH levels (42). This might reflect a common origin from neural crest cells associated with the multidirectional differentiation of tumor cells driven by *RET* mutation, presenting a unique molecular pathogenic mechanism of ECS.

The clinical presentation of hypercortisolism in most cases of MEN 2 varies, with differences in the rate of progression and the severity of ECS (13, 17). The clinical symptoms primarily include hypertension, hyperglycemia, muscle weakness, purple striae, and a cushingoid appearance. However, a significant proportion of cases lack the classic Cushing phenotype, which is not helpful in distinguishing between Cushing’s disease or pituitary MTC metastasis associated with MEN 2 (13, 50–53). Of the 14 patients, 93% exhibited no suppression with either low or high-dose dexamethasone, and neither of the remaining 2 patients responded to CRH, suggesting an ectopic source such as a MTC or PHEO. At this point, it is appropriate to consider that ECS may be caused by MEN 2, or at the very least, MEN 2 should be included in the differential diagnosis as a potential cause of ECS (14). Moreover, 64% of the 11 patients tested positive for ACTH or CRH immunostaining. However, CRH-negative evidence was not entirely absent in 36% of the patients, as indicated by abnormal proopiomelanocortin processing, increased secretion of ACTH precursors (24), or excessive secretion of ACTH and glucocorticoids due to positivity for CRF, urocortin 1, and urocortin 3 (42). Another possible explanation is that tumors exhibit high ACTH secretion but have low storage concentrations of ACTH within the tumor tissue, particularly in MTC (43%) (25, 54). However, beyond the ectopic production of ACTH, the possibility of ectopic production of CRH (22, 23, 27) or the co-secretion of CRH/ACTH cannot be excluded (30, 31, 37), nor can the presence of biphasic MTC and PHEO resulting in ECS (42). Therefore, a comprehensive approach that incorporates clinical information, biological tests, imaging scans, and immunohistochemistry is necessary for diagnosing ECS due to MEN 2 (14, 15, 17).

The management of ECS is often complex. Nonetheless, the goals of treatment are the resolution of hypercortisolism and control of the primary tumor (13, 14, 17). It should be noted, however, that the surgical complete removal of the ectopic CRH/ACTH source (aetiologic surgery), which leaves the patient free of the tumor, is rarely possible in advanced-MTC (14). In this review, only 36% experienced successful resolution of hypercortisolism through successive treatments, including total thyroidectomy with regional lymphadenectomy, and/or the use of metyrapone and selpercatinib, or ketoconazole, rather than a cure for MTC (22, 27, 32, 33). The primary therapeutic goal was indeed to prevent complications from hypercortisolemia and to improve the patient’s overall condition, thereby enabling systemic treatment for MTC to commence as soon as possible. Resolution of hypercortisolism can be achieved through medical treatment using adrenal steroidogenesis inhibitors (e.g., ketoconazole, metyrapone), adrenolytic agents (e.g., mitotane), and/or TKIs such as vandetanib, cabozantinib, sunitinib, sorafenib, and selpercatinib (21, 26, 29, 30, 32, 33). In fact, an additional 64% of these patients underwent BLA. Three of them received BLA as a rescue therapy after experiencing poor control of hypercortisolism with medical treatment (21, 26, 29, 30), while the remaining four, including our case, received it as first-line treatment for hypercortisolism (23, 24, 31). The current drug management of hypercortisolism is effective in the short term and sometimes in the long term. Adrenalectomy remains the most immediate and effective control measure, particularly in cases of uncontrolled hypercortisolism and its severe complications (31), concurrent adrenal disease (24), and the absence of a paraneoplastic ACTH secretion site (23). BLA has rapidly and safely improved ECS due to MEN 2-related MTC. BLA should always be considered a therapeutic option for rapid and effective control of hypercortisolism due to ECS, and laparoscopic BLA should be the preferred approach (36, 44, 55–57).

It is important to note that, unlike MTC, the efficacy of ECS resulting from PHEO is generally quite good, potentially due to the high rates of surgical cure and the infrequent occurrence of metastases in sporadic PHEO (15, 18). Unilateral adrenalectomy was performed on most patients, resulting in apparent cure, except in those with bilateral PHEO, where BLA were performed after treatment with an alpha1-adrenergic antagonist. As expected, six patients developed ECS due to PHEO, with a predominance of bilateral cases. Of these, 83% were cured of both PHEO and hypercortisolism through BLA, including one patient who underwent radical BLA with lymph node metastasectomy (40). Moreover, a patient was successfully treated for right-sided PHEO and hypercortisolism through a right adrenalectomy (41). This case underscores the need for clinicians to remain vigilant when managing both catecholamines and hypercortisolemia, as these conditions are synergistically complex and linked to multiple comorbidities, particularly elevating the risks of heart and metabolic diseases (e.g., arrhythmias, heart failure) (15, 18, 34, 35). Additionally, a considerable number of cases did not exhibit the typical Cushingoid features (for example, patient [21] developed ECS due to a right-sided PHEO, and patient [22] with ECS due to a biphasic MTC and PHEO). It is conceivable that performing adrenalectomy for PHEO without prior awareness of concurrent hypercortisolism (especially in those with unilateral PHEO, where contralateral adrenal atrophy is frequently observed) could induce a life-threatening adrenal crisis. The clinician should be aware of this possibility and be vigilant in recognizing and promptly managing glucocorticoid replacement therapy (15, 18, 21, 28, 38, 39, 42). Interestingly, none of these patients underwent adrenal-sparing surgery; all had total adrenalectomy (bilateral or unilateral). This may raise concerns that the former is difficult to correct hypercortisolism rapidly, pending further studies.

The presence of a patient with ECS due to biphasic MTC and PHEO who successively underwent BLA and a total thyroidectomy, achieving a good control of ECS. However, the option of a one-stage sequential PHEO-MTC and/or HPTH procedure in MEN2 with ECS (38, 40) and without ECS (2, 57) also has been reported. Nonetheless, preoperative cortisol-lowering therapy, correction of metabolic dysregulation (particularly hypokalemia), thromboprophylaxis, and antimicrobial treatment for opportunistic infections can be lifesaving, improve patients’ operative risk, and assist in preventing post-operative complications (15, 17, 19). It is crucial to emphasize that the timely implementation of the BLA should neither be delayed nor considered too late because of an overemphasis on surgical safety in cases of extremely severe and advanced cases of ECS (15, 17). Early and prompt diagnosis, particularly through integrated pedigree screening and predictive testing for *RET* mutations and serum Ctn levels, facilitates the implementation of individualized precision treatment for MEN2-related tumors. For instance, a total thyroidectomy, with or without neck lymph node dissection for MTC, and the timely cortisol-sparing adrenalectomy for PHEO, are crucial in reducing morbidity and mortality rates (1–3, 6, 7). In this review, three of the six deaths resulted from MTC progression, one died during laparotomy due to hypercortisolism, another due to complications of ECS, and one from myocardial ischemia.

We present a case that underscores the efficacy of emergency BLA in the immediate management of severe and refractory hypercortisolism, with a delayed diagnosis of ECS for 6 months. This case was thoroughly discussed in a multidisciplinary meeting. The patient’s condition improved markedly and rapidly post-operatively, allowing for the initiation of systemic therapy for advanced-MTC. Due to financial and other reasons, the patient subsequently underwent a clinical trial of a selective RET inhibitor with informed consent. Although targeted therapies such as vandetanib and selpercatinib, which have been reported to exert a direct antisecretory effect on neoplastic cells, can reverse hypercortisolism and sustain tumor burden control (13, 29, 30, 33, 58–60).

To our knowledge, this is the first report of a patient carrying the MEN 2A-harboring germline *RET*-p.C634Y mutation worldwide, and the first systematic review of the evidence for ECS caused by MEN 2-related MTC and/or PHEO. However, there are several limitations. First and foremost, all studies were either case reports or case series with relatively small numbers of patients. Secondly, the data was incomplete in its description (*RET* testing) and lacked systematic follow-up; none of the studies included a control group, making it challenging to assess treatment outcomes and prognosis. Thirdly, the studies included in this review span nearly seven decades, and there may be inherent reporting biases and heterogeneity, which limit the consistency of outcome comparisons. One method to address this issue is to establish a global, multicenter registry for more systematic reporting of cases.

## Conclusions

ECS secondary to MEN 2 presents additional challenges. While ECS in MEN2 is extremely rare, awareness of this association facilitates timely diagnosis and management, preventing delays and improving outcomes. Adrenalectomy typically achieves cure for the majority of ECS caused by PHEO, whereas most patients with advanced-MTC eventually require BLA to relieve their symptoms; however, the prognosis is generally poor. TKIs may have some efficacy in managing both MEN 2-related MTC and hypercortisolism due to ECS. The diagnosis and treatment of MEN 2-associated ECS necessitate integrated genetic testing, imaging localization, and multidisciplinary collaboration. Early identification of *RET* mutation types, precise management of hypercortisolism, and eradication of the primary tumor are essential to prevent complications and improve prognosis.

## Data Availability

The original contributions presented in the study are included in the article/**Supplementary Material**. Further inquiries can be directed to the corresponding author/s.

## Supplementary material

Supplementary Figure 1 Pre-operation CT imaging of the bilateral thyroid and CT imaging during ectopic Cushing’s syndrome. **(A)** CT scans revealed bilateral thyroid masses (left, 2.3 cm; right, 4.8 cm). **(B)** Contrast-enhanced imaging reveals significant heterogeneous enhancement with multiple hypodense areas within the lesion. **(C, D)** The right neck and upper mediastinum exhibited an irregular mass-like soft tissue density lesion measuring approximately 6.6 cm × 4.4 cm × 4.1 cm, with indistinct borders **(E, F)**. Contrast-enhanced imaging showed significant heterogeneous enhancement, with multiple hypodense areas within the lesion. The adjacent trachea was slightly compressed and deviated to the left. Scattered multiple lymph node shadows were observable in the bilateral neck, supraclavicular region, and upper mediastinum, with some of them enlarged. Contrast-enhanced imaging revealed marked heterogeneous enhancement, especially in the larger level III lymph nodes.

Supplementary Figure 2 The immunohistochemical staining presentation of the recurrence of medullary thyroid carcinoma. **(A)** Medullary thyroid carcinoma cell negative for corticotrophin-releasing hormone (CRH) staining (CRH, × 200); **(B)** Medullary thyroid carcinoma cell weakly positive for cytoplasmic adrenocorticotropic hormone (ACTH) staining (ACTH, × 200).

Supplementary Figure 3 Histopathological features of the adrenal cortex hyperplasia. **(A)** The adrenal cortex thickening, accompanied by hyperplasia of the zona glomerulosa and zona fasciculata, predominantly in the zona fasciculata, with clear boundaries and no capsule. Nodular hyperplasia of the left adrenal medulla was visible. The medullary cells were large, with abundant cytoplasm, amphochromasia, round nuclei, and prominent nucleoli (haematoxylin-eosin; original magnification, × 200). **(B)** Nodular hyperplasia of the left adrenal medulla (haematoxylin-eosin; original magnification, × 2).
