# Supplementary figures and images for: Presentation of multiple endocrine neoplasia type 2A-associated ectopic cushing’s syndrome: case report and a systematic review
Source: Front Endocrinol (Lausanne). 2025 Nov 11;16:1644751. doi: 10.3389/fendo.2025.1644751 (PMC12645412; doi:10.3389/fendo.2025.1644751)

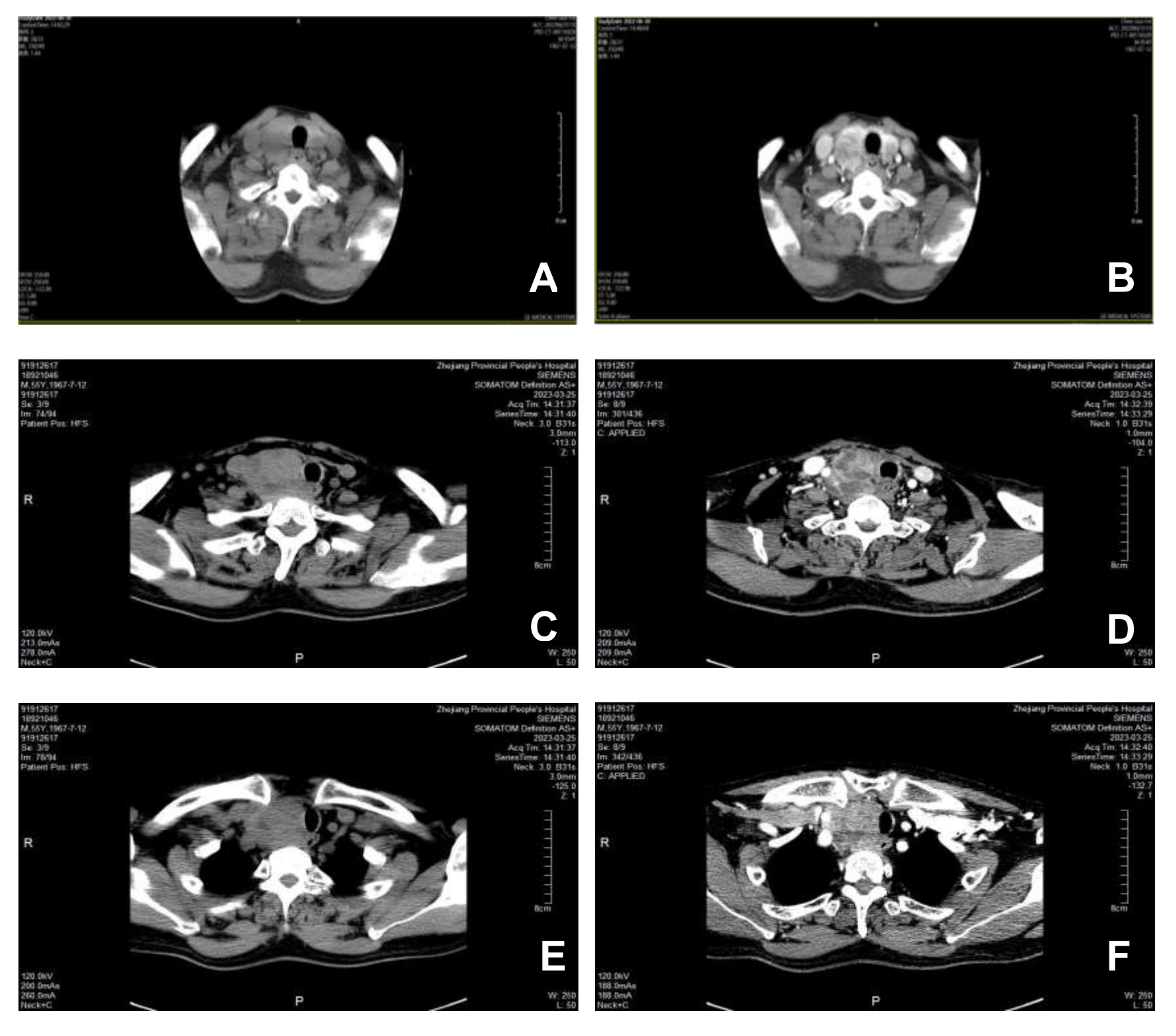

Supplement: Supplementary Figure 1 — Pre-operation CT imaging of the bilateral thyroid and CT imaging during ectopic Cushing’s syndrome. (A) CT scans revealed bilateral thyroid masses (left, 2.3 cm; right, 4.8 cm). (B) Contrast-enhanced imaging reveals significant heterogeneous enhancement with multiple hypodense areas within the lesion. (C, D) The right neck and upper mediastinum exhibited an irregular mass-like soft tissue density lesion measuring approximately 6.6 cm × 4.4 cm × 4.1 cm, with indistinct borders (E, F). Contrast-enhanced imaging showed significant heterogeneous enhancement, with multiple hypodense areas within the lesion. The adjacent trachea was slightly compressed and deviated to the left. Scattered multiple lymph node shadows were observable in the bilateral neck, supraclavicular region, and upper mediastinum, with some of them enlarged. Contrast-enhanced imaging revealed marked heterogeneous enhancement, especially in the larger level III lymph nodes. [file Image1.tiff]

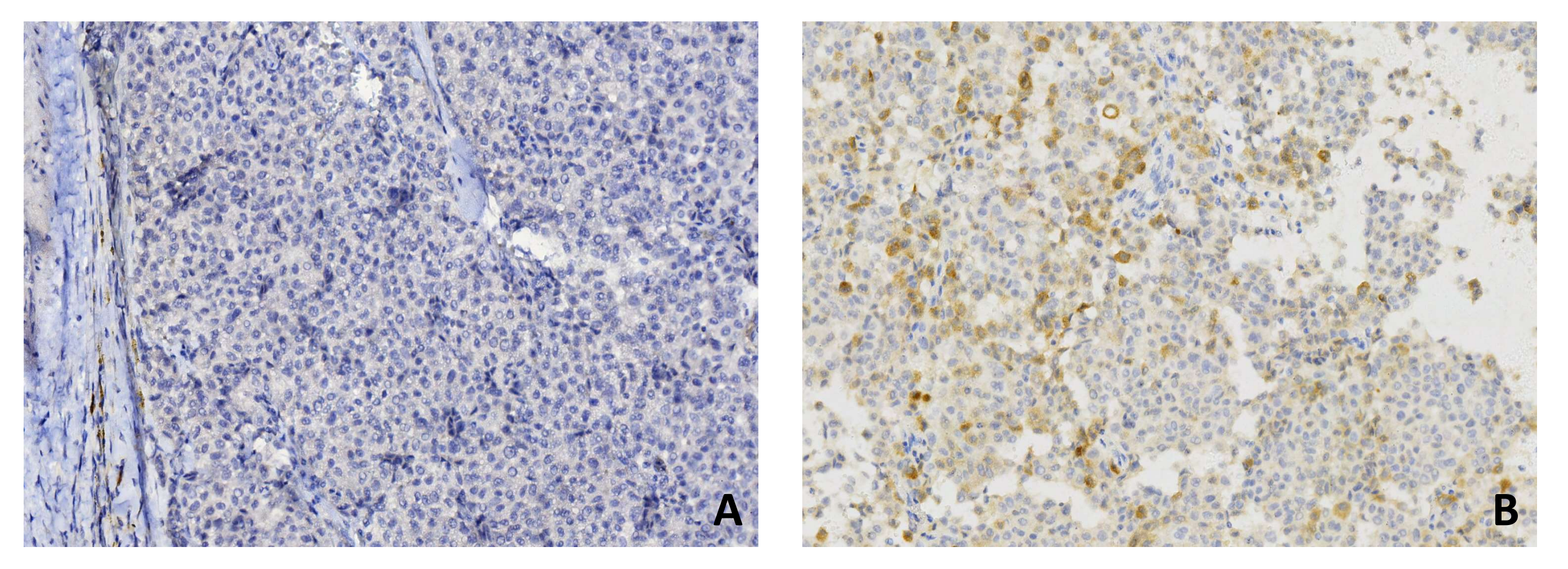

Supplement: Supplementary Figure 2 — The immunohistochemical staining presentation of the recurrence of medullary thyroid carcinoma. (A) Medullary thyroid carcinoma cell negative for corticotrophin-releasing hormone (CRH) staining (CRH, × 200); (B) Medullary thyroid carcinoma cell weakly positive for cytoplasmic adrenocorticotropic hormone (ACTH) staining (ACTH, × 200). [file Image2.tiff]

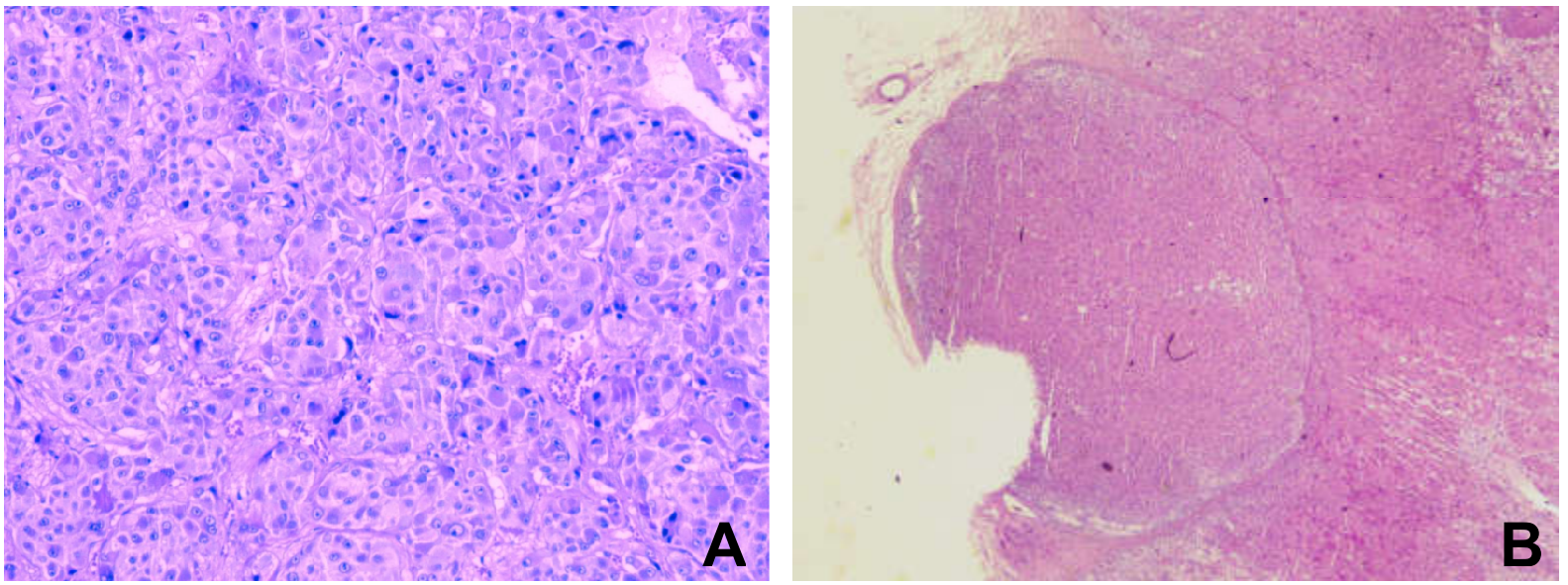

Supplement: Supplementary Figure 3 — Histopathological features of the adrenal cortex hyperplasia. (A) The adrenal cortex thickening, accompanied by hyperplasia of the zona glomerulosa and zona fasciculata, predominantly in the zona fasciculata, with clear boundaries and no capsule. Nodular hyperplasia of the left adrenal medulla was visible. The medullary cells were large, with abundant cytoplasm, amphochromasia, round nuclei, and prominent nucleoli (haematoxylin-eosin; original magnification, × 200). (B) Nodular hyperplasia of the left adrenal medulla (haematoxylin-eosin; original magnification, × 2). [file Image3.tiff]
