# Supplementary material for: Presentation of multiple endocrine neoplasia type 2A-associated ectopic cushing’s syndrome: case report and a systematic review
Source: Front Endocrinol (Lausanne). 2025 Nov 11;16:1644751. doi: 10.3389/fendo.2025.1644751 (PMC12645412; doi:10.3389/fendo.2025.1644751)
Supplement: Supplementary file 4 [file Table1.doc]

Supplementary

Table S1 Clinical characteristics of the RET p.C634Y mutation carriers with MEN2A and associated ECS

| **Individual** | **Gender**  **(M/F)** | **Age**  **(year)** | **Age at diagnosis (yr)** | | | | **Pre-/Post Ctn (pg/mL)** | **MTC (Bi)** | | | **PHEO** | | **PHPT** | | **Follow-up**  **(months) *** |
| --- | --- | --- | --- | --- | --- | --- | --- | --- | --- | --- | --- | --- | --- | --- | --- |
|  |  |  | **MTC** | **PHEO** | **PHPT** | **ECS** |  | **Surgery** | **Max size**  **(cm)** | **TNM** | **Surgery** | **Max size**  **(cm)** | **Surgery** | **Max size**  **(cm)** |  |
| Ⅱ-3 | F | 76 | 75 | 75 | NA | – | >2,000 / NA | Rejected | 2.2 × 2.0 | cT2NxMx | Rejected | 1.5 × 1.2 (L) | NA | NA | NA |
| Ⅲ-2 | M | 59 | 55 | 59 | 55 | – | >2,000 / < 0.5 | TT + BiLND(VI) + MBiND | 2.3 × 2.2 | T2N1bM0 | ASS (L) | 1.6 × 1.5 (L) | PAE | 1.7 × 1.5 (L) | 6 |
| **Ⅲ-3 ^†^** | M | 56 | 54 | 55 | – | 55 | >2,000 / >2,000 | TT + BiLND(VI) + MBiND + MLD | 4.8 × 3.3 | T3aN1bM1 | ^#^ BLA | 1.2 × 1.1 (Bi) | – | – | 12 |
| Ⅲ-7 | F | 55 | 51 | 51 | – | – | >2,000 / < 0.5 | TT + BiLND(VI) | 3.7 × 2.1 | T2N0M0 | ASS (R ) | 4.5 × 2.8 (R) | – | – | 51 |
| Ⅲ-8 | M | 51 | 51 | 41 | NA | – | 127.05 / NA | Rejected | NA | NA | ASS (R ) | 3.2 × 2.5 (R) | NA | NA | 120 |
| Ⅲ-9 ^#^ | F | 49 | 48 | 48 | – | – | 69.27 / < 0.5 | TT + BiLND(VI) | 1.3 × 0.8 | T1aN0M0 | ASS (L) | 1.2 × 0.9 (L) | *–* | *–* | 10 |
| IV-1  (proband ) | M | 35 | 30 | 35 | *–* | *–* | 1088.0 / 2.7 | TT + BiLND(VI) + MLND | 1.8 × 1.3 | T1bN1bM0 | ASS (R) | 2.1 × 1.5 (R) | *–* | *–* | 7 |
| IV-9 | F | 20 | NA | NA | NA | – | NA | Rejected | NA | NA | NA | NA | NA | NA | NA |
| V-1 | F | 4.5 | – | – | – | – | 2.02 / NA | WW | – | NA | – | – | – | – | 6 |

MEN2A, multiple endocrine neoplasia type 2; **^†^**, ECS, ectopic Cushing’s syndrome; M/F, male/female; MTC, medullary thyroid carcinoma; PHEO, pheochromocytoma; PHPT, primary hyperparathyroidism; pre/post-Ctn, pre/post-operative basal serum calcitonin (normal male < 8.4 pg/mL and female < 5.0 pg/mL); Max size, maximum size (all initial); TNM, tumor-node-metastasis (tumor stage); R, right; L, Lift; Bi, bilateral; TT, total thyroidectomy; LND(VI), level VI lymph node dissection; MBiND, modifed bilateral neck dissection; MR(L)ND, modifed right (left) neck dissection; MLD, mediastinal lymphatic dissection; ASS, adrenal-sparing surgery; PAE, parathyroid adenoma excision; BLA, bilateral adrenalectomy; WW, watchful waiting; NA, not available; –, negative.

^*^, after the final MEN2A-related surgery or start watchful waiting.

**^†^,** patients presented MEN 2 with ECS.

**^#^,** patients with successive ASS and thyroidectomy in a single procedure.
