# Supplementary material for: Presentation of multiple endocrine neoplasia type 2A-associated ectopic cushing’s syndrome: case report and a systematic review
Source: Front Endocrinol (Lausanne). 2025 Nov 11;16:1644751. doi: 10.3389/fendo.2025.1644751 (PMC12645412; doi:10.3389/fendo.2025.1644751)
Supplement: Supplementary file 5 [file Table2.doc]

| ***Serum/plasma***  ***biochemical markers*** | ***Pre-operation*** | ***Post-operation (8:00 am)*** | | | | ***reference value*** |
| --- | --- | --- | --- | --- | --- | --- |
|  |  | ***1 months*** | ***3 months*** | ***6 months*** | ***12 months*** |  |
| Blood pressure (mmHg) | 200/95 | 100/80 | 127/64 | NA | 122/90 | 90~139/60~89 |
| BMI (kg/m^2^) | 24.02 | NA | NA | NA | 19.9 | 18.5~23.9 |
| Calcitonin (pg/mL) | >2000 | ＞2000 | ＞2000 | ＞2000 | ＞2000 | < 8.4 |
| Carcinoembryonic antigen (ng/mL) | 70.8 | 77.1 | 150.1 | 500.0 | 880.5 | < 5.0 |
| Parathyroid hormone (pg/mL) | 6.0 | 1.4 | NA | NA | <5.4 | 15–65 |
| Metanephrine (pg/mL) | 79.7 | NA | NA | NA | 14.88 | <62.0 |
| Normetanephrine (pg/mL) | 90.7 | NA | NA | NA | 91.83 | <145.0 |
| 3-Methyltyramine (pg/mL) | 2.4 | NA | NA | NA | 1.03 | <18.4 |
| Aldosterone (pg/mL) | 8.2 | NA | NA | NA | 62.76 | 70-300 |
| ACTH (pg/mL) | 189.0 at 8:00  125.0 at 16:00  165.0 at 24:00  *183.0 at 8:00 | 220.0 | 731.0 | NA | 649.58 | < 46 |
| Cortisol (μg/L) | 429.30 at 8:00  408.14 at 16:00  446.61 at 24:00  *538.27 at 8:00 | NA | 194.40 | NA | 327.89 | 67-226 (8:00am)  <100 (4:00p.m.) |
| Potassium (mmol/L) | 2.40 | 3.64 | 4.56 | 4.49 | 4.46 | 3.5-5.3 |
| Calcium (mmol/L) | 1.70 | 2.34 | 2.37 | 1.89 | 1.67 | 2.10-2.80 |
| Fasting glucose (mmol/L) | 12.7 | 4.88 | 4.42 | 4.23 | 4.2 | 3.89-6.11 |
| Glycosylated hemoglobin (%) | 8.2 | 4.9 | NA | NA | 4.9 | < 6 |

Supplementary Table S2. Laboratory data before and after bilateral adrenalectomy

^*^After 1 mg overnight dexamethasone suppression test.
